# Stem/progenitor cells in normal physiology and disease of the pancreas

**DOI:** 10.1016/j.mce.2021.111459

**Published:** 2021-12-01

**Authors:** Mario Enrique Alvarez Fallas, Sergio Pedraza-Arevalo, Ana-Maria Cujba, Teodora Manea, Christopher Lambert, Rosario Morrugares, Rocio Sancho

**Affiliations:** a Centre for Stem Cells and Regenerative Medicine, Faculty of Life Sciences & Medicine, King's College London, London, UK; b Department of Medicine III, University Hospital Carl Gustav Carus, Dresden, Germany; c Instituto Maimonides de Investigacion Biomedica de Cordoba (IMIBIC), Cordoba, Spain; d Departamento de Biologia Celular, Fisiologia e Inmunologia, Universidad de Cordoba, Cordoba, Spain; e Hospital Universitario Reina Sofia, Cordoba, Spain

**Keywords:** Pancreas progenitors, Stem cells, Cell fate decisions, scRNA-seq

## Abstract

Though embryonic pancreas progenitors are well characterised, the existence of stem/progenitor cells in the postnatal mammalian pancreas has been long debated, mainly due to contradicting results on regeneration after injury or disease in mice. Despite these controversies, sequencing advancements combined with lineage tracing and organoid technologies indicate that homeostatic and trigger-induced regenerative responses in mice could occur. The presence of putative progenitor cells in the adult pancreas has been proposed during homeostasis and upon different stress challenges such as inflammation, tissue damage and oncogenic stress. More recently, single cell transcriptomics has revealed a remarkable heterogeneity in all pancreas cell types, with some cells showing the signature of potential progenitors. In this review we provide an overview on embryonic and putative adult pancreas progenitors in homeostasis and disease, with special emphasis on *in vitro* culture systems and scRNA-seq technology as tools to address the progenitor nature of different pancreatic cells.

## Highlights

• The existence of putative progenitors beyond the embryonic stage capable of contributing to pancreas homeostasis has been suggested.
• Heterogeneity is observed in the different compartments in the pancreas.
• Tumours in the pancreas arise from different originating cells that determine tumour evolution and prognosis.
• Pancreatic organoid cultures provide a platform to study embryonic and adult progenitors and to explore new ways of generating new β-cells.
• Single cell transcriptomics allowed the prediction of multipotent cell populations within the adult human and murine pancreas.

## 1 Introduction

**Fig. 1:**
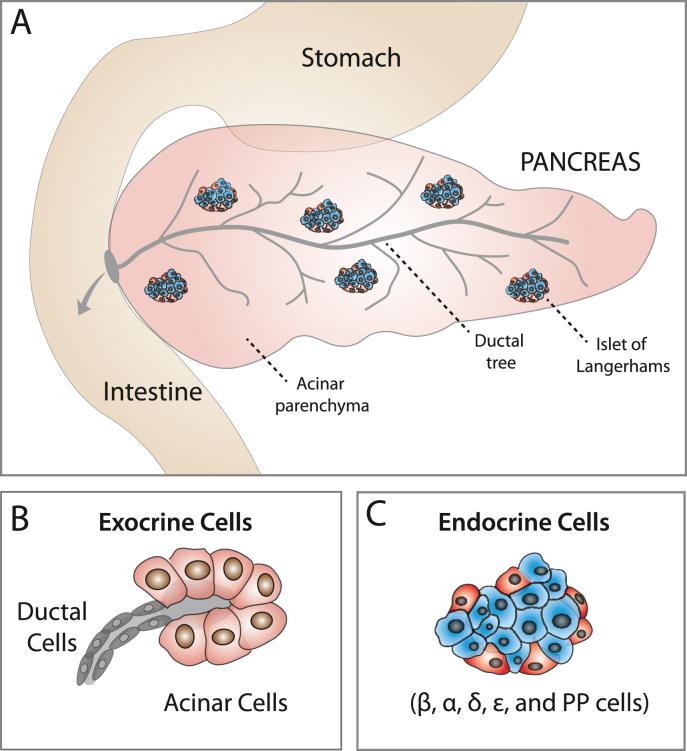
**The endocrine and exocrine pancreas: (A) The pancreas contains an exocrine and an endocrine compartment.** The exocrine compartment is composed of the pancreatic ductal network and acini, making up 98–99% of the pancreas. The endocrine compartment contains the islets of Langerhans which only accounts for 1–2% of pancreatic cells. **(B) Exocrine cells.** The exocrine compartment comprises the ductal cells, which form the pancreatic ductal network, and the acinar cells, which form the pancreatic acini. **(C) Endocrine cells.** The endocrine compartment of the pancreas contains multiple types of endocrine cells, such as α, β, δ, ε and PP cells, organised in pancreatic islets.

The pancreas consists of an abundant exocrine compartment (98–99% of the pancreas mass) composed of pancreatic ducts and acini, and a smaller endocrine compartment, restricted to the islets of Langerhans (1–2% of the pancreas mass) (Fig. 1). Both endocrine and exocrine cells differentiate from a common progenitor during embryonic development. Ductal and acinar cells fulfil the digestive functions of the gland by synthesizing and releasing enzymes to the duodenum. The islets of Langerhans regulate glucose homeostatic functions by releasing insulin, glucagon and somatostatin hormones secreted by β-, α- and δ-cells respectively (Edlund, 2002) (Fig. 1). Among the endocrine cells, β-cells have been the main focus of pancreas research due to their involvement in diabetes. Loss of β-cells or β-cell functionality is the cause of hyperglycaemia in type 1 and type 2 diabetes. While diabetes is a treatable disease, it still has no long-term curative remedy (Tan et al., 2019). This is in part due to the inability of the adult pancreas to regenerate lost or defective β-cells.

Despite the permanent loss of β-cell function in diabetes and the relatively low turnover of β-cells during adulthood, evidence in mice suggests that the adult pancreas has a certain degree of plasticity, allowing for limited regeneration when provided with specific stimuli. Although the existence of true progenitor cells is still a matter of debate (reviewed in Bonner-Weir et al., 2010; Demcollari et al., 2017; Dominguez-Bendala et al., 2019; Zhou and Melton, 2018), progenitor-like cells within the different compartments of the pancreas have been described after physiological, stress-induced and oncogenic challenges, suggesting that a pool of stem/progenitor cells could exist in the pancreas beyond the embryonic stage. The ability to expand pancreatic cells *ex vivo*, combined with new technologies, such as single cell RNA-seq (scRNA-seq), have provided support for the existence of such putative adult pancreatic progenitors (Grun et al., 2016; Muraro et al., 2016; Wang et al., 2020). In this review we will provide an overview of progenitor-like functions of the embryonic and adult pancreas in homeostasis and disease, with special emphasis on the latest *ex vivo* culture systems and scRNA-seq technologies exploring the progenitor nature of different pancreatic cells.

## 2 Embryonic progenitors during pancreas development

**Fig. 2:**
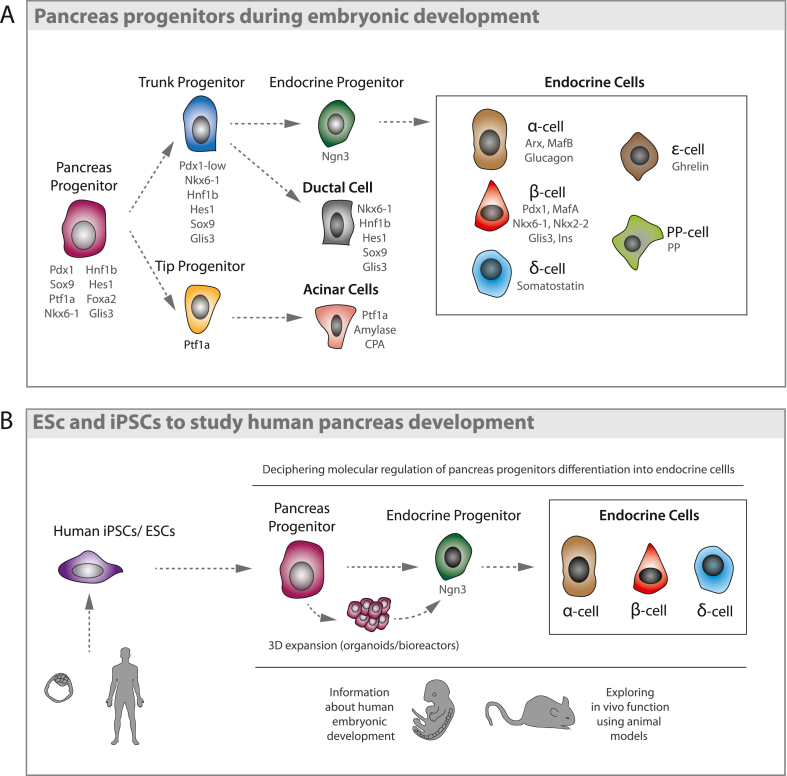
**Pancreas development: (A) Pancreas progenitors during embryonic development.** Pdx1+ pancreatic progenitors give rise to the three differentiated cell types in the adult pancreas. Maintenance of Ptf1a expression specifies the acinar cell lineage in tip progenitors, while trunk progenitors maintain Nkx6.1 expression. While most trunk progenitors generate ductal cells, transient Ngn3 expression in some trunk progenitors induces an endocrine fate, generating α, β, δ, ε and PP cells. **(B) ESCsand iPSCs to study human pancreatic development.** Human-derived ESCs and iPSCs can be expanded and successfully differentiated, first to pancreatic progenitors and then to the endocrine cell types found in the human pancreas. Elucidating the molecular mechanisms that regulate their differentiation *in vitro* can provide insight into the embryonic development of the pancreas, while also opening new avenues for future therapies.

The three differentiated adult pancreatic cell types, acinar, ductal and endocrine cells, arise from a common Pdx1+ progenitor during embryonic development (Edlund, 2002). Ablation of Pdx1 leads to pancreatic agenesis (Offield et al., 1996; Stoffers et al., 1997), highlighting the importance of this transcription factor in giving rise to both exocrine and endocrine cells. In the mouse, Pdx1+Sox9+Ptf1a+ tripotent progenitors are found in the posterior foregut endoderm starting from embryonic day E8.5 (Human Carnegie stage 4). Further maturation into the pancreatic bud leads to the secondary transition (E12-E15), when differentiating cells assume a ductal, acinar or endocrine identity via a fine-tuned signaling network (Edlund, 2002) (Fig. 2A). While tripotent progenitors now co-express Nkx6.1, as the development of the pancreatic epithelium progresses, the progenitors at the peripheral “tips” domain start losing its expression, giving rise to acinar cells. Conversely, progenitors retaining Nkx6.1 in the “trunk” domain will lose Ptf1a expression and form islets and pancreatic ducts respectively (Schaffer et al., 2010) (Fig. 2A).

Both Ptf1a and Nkx6.1 are essential not only during embryogenesis, but also in adult cell fate maintenance. In humans, nonsense mutations in Ptf1a lead to exocrine pancreatic agenesis and neonatal diabetes (Krapp et al., 1998), while reduced Ptf1a dosage results in pancreatic hypoplasia, insufficient insulin secretion and glucose intolerance in mice (Fukuda et al., 2008). Similarly, Nkx6.1 inactivation in adult mice causes rapid-onset diabetes and hypoinsulinemia (Taylor et al., 2013), whilst some of its variants have been associated with Type 2 diabetes in genome-wide association studies (Yokoi et al., 2006). Indeed, reduced Nkx6.1 expression is observed during the onset of Type 2 diabetes in humans (Guo et al., 2013).

While the majority of the progenitor cells in the pancreatic “trunk” domain maintain expression of factors such as Hnf6, Hnf1β, Sox9 and Glis3 (Jacquemin et al., 2000; Kang et al., 2009; Kropp et al., 2019; Shih et al., 2012) to form ductal structures, some cells start expressing Neurogenin-3 (Ngn3), becoming endocrine progenitors (Gradwohl et al., 2000) (Fig. 2A). Ngn3 transcripts can be detected as early as E8.5 during mouse pancreas development; however, most endocrine precursors are generated as a result of a second wave of Ngn3 expression, with absolute numbers peaking at E15.5 (Villasenor et al., 2008). Ngn3+ endocrine progenitors generate all five types of endocrine cells found in pancreatic islets (Gu et al., 2002; Heller et al., 2005): glucagon-secreting α-cells, insulin-secreting β-cells, somatostatin-secreting δ-cells, ghrelin-secreting ε-cells and PP cells, which secrete pancreatic polypeptide. Ngn3 is required for the activation of endocrine differentiation as all the different Ngn3 knockout models (mouse and pig) generated, resulted in perinatal lethality due to a lack of insulin, glucagon and somatostatin-producing cells in the pancreas (Sheets et al., 2018).

Once the endocrine fate is activated, expression of the transcription factor Arx pushes endocrine cells towards an α-cell fate, while Pax4 is required for β-cell generation (Collombat et al., 2003) (Fig. 2A). Pax4 and Arx expression levels are also essential for maintenance of β-cells and α-cells respectively during adulthood, as Pax4 overexpression in α-cells, as well as Arx loss, induces α-cell-to-β-cell plasticity (Collombat et al., 2009; Courtney et al., 2013). Moreover, loss of both Pax4 and Arx leads to an increase in the δ-cell population (Collombat et al., 2005), suggesting that δ-cells could represent an endocrine ground cell state.

Playing an important role in the maturation of pancreatic endocrine cells, the transcription factors MafA and MafB are expressed at a later stage during development (Hang and Stein, 2011). In mice, MafB is expressed in both immature α- and β-cells, and is required for α-cell maturation, playing an important role in glucagon production (Artner et al., 2006). MafA is instead exclusively expressed in β-cells (Artner et al., 2010; Nishimura et al., 2015), although during pregnancy, a small fraction of β-cells re-express MafB (Pechhold et al., 2009). In humans, co-expression of MAFB with MAFA is observed in mature β-cells (Dai et al., 2012). Cyphert and colleagues have recently shown that the mouse homodimerized MafA might be the equivalent of the heterodimer MAFA/B in humans, and that MAFA dynamics differ from juvenile age through adulthood (Cyphert et al., 2019). The timing of MAFA expression is indeed essential, as premature MafA expression in Ngn3+ precursors impairs differentiation and hormone secretion in mice (He et al., 2014). Coherently, mutual deletion of the two transcription factors revealed that MafA has a greater impact on β-cell activity and islet morphology (it is also known as a Maturity-Onset Diabetes of the Young or MODY gene) (Hang et al., 2014), while MafB deletion results mainly in an alpha cell phenotype (Conrad et al., 2016). While reportedly important for human β-cell maturation and development, the MAFA/B mechanism in the human pancreas is not yet fully understood (Riedel et al., 2012).

Through the knowledge acquired on pancreas embryogenesis, successful culture and differentiation methods for pluripotent stem cells (embryonic and induced) have been achieved, accurately recapitulating the different embryonic stages (Fig. 2B). Human-derived ESCs and iPSCs can be expanded and differentiated, first to pancreatic progenitors and then to the endocrine cell types found in the human pancreas (D'Amour et al., 2006; Kroon et al., 2008; Pagliuca et al., 2014; Rezania et al., 2014; Rezania et al., 2012; Russ et al., 2015). Elucidating the molecular mechanisms that regulate their differentiation *in vitro* can provide insights into embryonic progenitors, while also opening new avenues for future therapies.

However, while progenitors during embryonic development are well characterised, the existence of progenitors in the postnatal pancreas remains controversial.

## 3 Progenitors and plasticity in the adult pancreas

As opposed to highly proliferative tissues such as skin and intestine, the regenerative capacity of the pancreas is very limited. The conventional view is that cell proliferation is the only source of this limited regeneration, occurring independently within the endocrine and exocrine compartments (Georgia and Bhushan, 2004; Kopinke et al., 2011; Saisho et al., 2013; Sangiorgi and Capecchi, 2009). The existence of an upstream stem or progenitor cell playing a role in the maintenance of the mature organ has been fundamentally questioned (Dor et al., 2004; Kopp et al., 2011; Solar et al., 2009). By lineage tracing β-cells using RIP–CreER; Z/AP mice, Dor and colleagues demonstrated that pre-existing β-cells are the major source of new β-cells in adulthood and after pancreatectomy in mice, suggesting that terminally differentiated β-cells retain a significant proliferative capacity *in vivo* (Dor et al., 2004). Additionally, in later works using ductal cell mouse tracing models Sox9-CreERT and Hnf1β-CreERT, no endocrine tracing was observed in homeostatic or challenge conditions, though the efficiency of ductal tracing was far from complete (Kopp et al., 2011; Solar et al., 2009).

Either by replication or by other mechanisms, islet regeneration is rare under normal conditions. In mice, the capacity for β-cell proliferation has been shown during pregnancy, where preexisting β-cells expand to meet the increased demand for insulin (Karnik et al., 2007; Kim et al., 2010; Sorenson and Brelje, 1997). Replication has been also observed upon metabolic stress or by high fat diet (Cox et al., 2016; Kahn et al., 2006). In humans, evidence suggests that obesity induces an increase in β-cell replication (Butler et al., 2010; Saisho et al., 2013).

**Fig. 3:**
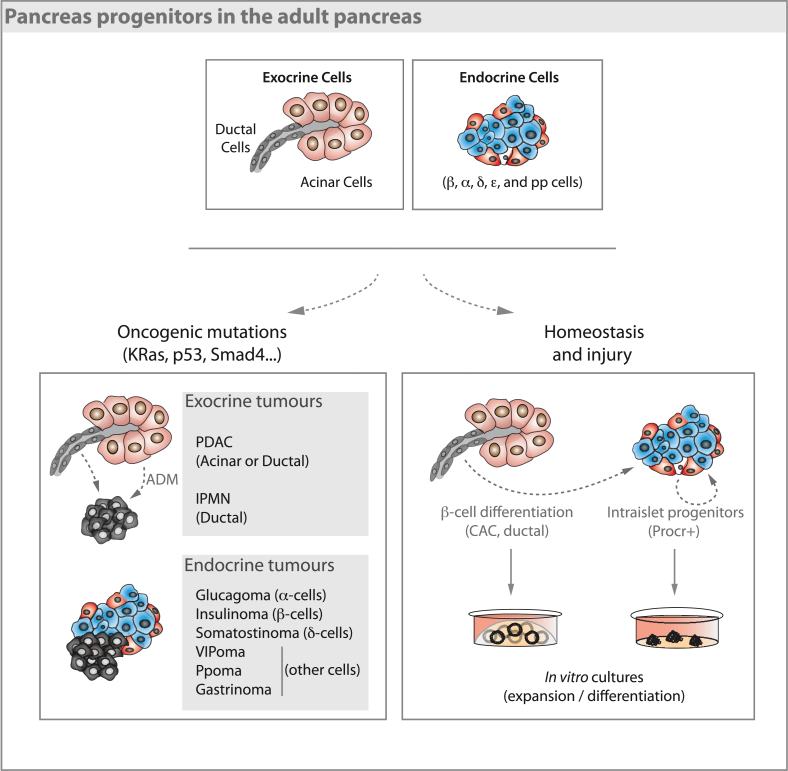
**Pancreas progenitors in the adult pancreas.** Both exocrine and endocrine cells in the adult pancreas retain some plasticity when challenged with different triggers (oncogenic mutations, injury). Although the cell of origin for the different types of endocrine tumours is still unclear, ductal and acinar progenitor cells can become neoplastic after undergoing oncogenic mutations to generate PDACs or IPMN. During homeostasis and upon some injuries centro acinar cells (CAC), ductal cells and Procr+  intraislet progenitors can differentiate to β-cells both *in vivo* and *in vitro*.

In addition to replication, δ-to β-cell conversion has been reported in diabetic mice (Chera et al., 2014), opening the possibility that newly formed islets (or β-cells) could also arise from cell conversion. Indeed, the fact that terminally differentiated cells in the pancreas can give rise to other pancreatic cell types has been suggested not only by using challenging stimuli in mice (Gu et al., 2002; Md Moin et al., 2016; Tellez and Montanya, 2014; Xu et al., 2008), but also by modern techniques such as scRNA-seq analysis (Baron et al., 2016; Muraro et al., 2016). Several works over the last years have revealed a remarkable heterogeneity of gene expression within different pancreatic cell types (Benninger and Hodson, 2018; Razavi et al., 2015; Stanescu et al., 2017), and it has been suggested that some pancreatic subpopulations could act as facultative progenitors (Beamish et al., 2016; Beer et al., 2016) (Fig. 3). In a recent work, using the Aldh1b1-CreERT; R26-tdTomato mouse model, where CreERT is under the control of the centroacinar cell marker Aldh1b1, tdTomato labelling was observed in ducts, islets and acinar cells 24 weeks post-tamoxifen injection, reaching almost 0.6% of labelling within the β-cell compartment (Mameishvili et al., 2019), suggesting that centroacinar cells could contribute to β-cell homeostasis.

A surprising level of plasticity can be induced in the different pancreatic cell types upon different challenging and/or artificial stimuli (Chera et al., 2014; Li et al., 2010; Sancho et al., 2014; Storz, 2017; Wang et al., 1993; Zhou et al., 2008). Forced expression of Pdx1, Ngn3 and MafA, all of which play an essential role in endocrine specification and β-cell maturation (Fig. 2), has been shown to be sufficient for successful reprogramming of acinar cells (Li et al., 2014; Zhou et al., 2008) to β-like cells. Interestingly, adult ductal cells seem to acquire a progenitor state in response to injury, implying that they might regain endocrine and exocrine differentiation potential (Bonner-Weir et al., 1993; Li et al., 2010). In line with this, pancreatic duct ligation (Bonner-Weir et al., 2008; Xu et al., 2008), overexpression of TGFα in ductal cells (Wang et al., 1993), pancreatic ductal deletion of Fbw7 (Sancho et al., 2014), and Pax4 overexpression in both α-cells (Al-Hasani et al., 2013) and δ-cells (Druelle et al., 2017) have uncovered a potential for transdifferentiation of these cells towards β-cells (Fig. 3). Furthermore, it has been shown that inflammatory cytokines are able to induce endocrine differentiation of ductal cells (Valdez et al., 2016), as well as acinar cells (Lemper et al., 2015), by activating Stat3. More recent findings suggest that many of the cell types found in the pancreas show a degree of plasticity when subjected to specific triggers, being able to adapt to changes in insulin demand. For example, glucocorticoid-induced insulin resistance was shown to induce a massive increase in β-cell mass (Courty et al., 2019). A fasting-mimicking diet has also been shown to induce an embryonic development gene expression program in adult β-cells, resulting in Ngn3-mediated β-cell neogenesis. Interestingly, repeated cycles of fasting-mimicking diet were able to reverse β-cell failure and rescue mice from Type 1 and Type 2 diabetes (Cheng et al., 2017). Though these works suggest the existence of putative adult pancreas progenitors, seminal lineage tracing experiments in mice (Dor et al., 2004; Kopp et al., 2011; Solar et al., 2009) have produced contradicting results, raising the question of whether the specificity and label efficiency of the tracing could provide a layer of inaccuracy (reviewed in Dominguez-Bendala et al., 2019).

In addition to ductal cells, β-cells themselves have shown a great degree of heterogeneity, including cell complexity in terms of organelles and granules (Katsuta et al., 2012) and glucose response sensitivity and processing (Benninger and Piston, 2014; Kiekens et al., 1992; Van Schravendijk et al., 1992), besides their molecular signature (Dominguez-Gutierrez et al., 2019). Interestingly, Procr+ cells, identified after a thorough scRNA-seq analysis of murine islets, were able to give rise to new α-, β- and δ-cells during homeostasis. Procr+ cells were characterised by a transcriptional signature indicative of epithelial-to-mesenchymal transition as well as the absence of terminally differentiated endocrine and exocrine pancreatic cell markers. This work provides evidence for a new mechanism of β-cell neogenesis in normal physiological conditions (Wang et al., 2020).

Nevertheless, the capacity of normal β-cells to proliferate is still considered to account for most of the β-cell neogenesis that occurs during normal pancreatic homeostasis (Klochendler et al., 2016). The low numbers and technical difficulties detecting cells with progenitor features being one of the reasons of progenitor skepticism.

## 4 Pancreas plasticity and tumour formation

It has been classically assumed that any cell of the body may undergo oncogenic changes to become tumorigenic and form a tumour. However, increasing evidence of stem-like features of tumoral cells has opened a debate of whether tumours are originated by stem cells progenitors or by de-differentiated cells. On the one hand, the high differentiation of some low aggressive tumours alongside the capacity of differentiated cells to become stem-like in normal tissues, point to the possibility of a de-differentiation process in tumorigenesis (Carvalho, 2020; Gabbert et al., 1985; Zhou et al., 2019). On the other hand, plenty of evidence suggests that stem or progenitor cells are the ones most likely to accumulate all the necessary changes to produce a tumour (Hata et al., 2018; Prager et al., 2019; Sell, 2010).

In the pancreas, tumours are classified in two groups depending on whether they arise in the exocrine or in the endocrine tissue (Fig. 3). Tumours from the exocrine compartment are the most common and among them, adenocarcinoma is the most frequent pancreatic tumour (Rawla et al., 2019). This type of neoplasia arises from the acini or from the epithelial cells of the pancreatic ducts, so they are called pancreatic ductal adenocarcinoma (PDAC). PDAC represents around 90% of all pancreatic cancer cases (Rawla et al., 2019) and are the most aggressive type of pancreatic tumour, with a high metastatic rate and a five-year survival rate of barely 8% (Siegel et al., 2018; Werner et al., 2013). Different types of PDAC precursor lesions have been described: pancreatic intraepithelial neoplasia (PanIN), intraductal papillary mucinous neoplasia (IPMN), intraductal tubular papillary neoplasm (ITPN) and pancreatic mucinous cystic neoplasm (MCN), which are associated with different PDAC prognosis (Riva et al., 2018). However, it is not yet clear whether all these precursor lesions arise from different originating cells.

Acinar cells are known to have a degree of plasticity able to partially regenerate the pancreas through a process called acinar to ductal metaplasia (ADM) (Kopp et al., 2012) (Fig. 3). After certain types of stress, such as tissue damage or inflammation, acinar cells acquire a ductal-like phenotype with progenitor-like properties (Gidekel Friedlander et al., 2009). Although this process is much more common in pancreatitis, some progenitor-like ADM cells acquire activating mutations in the KRAS proto-oncogene and become PanINs (Kanda et al., 2012). This step is generally considered the first one in PDAC development, which progresses further by inactivation of different tumour suppressor pathways (Guerra et al., 2007; Kanda et al., 2012). Additionally, Aldh1b1+ expression has also been suggested to be linked to PDAC initiation (Mameishvili et al., 2019).

Interestingly, while either acinar or ductal cells have been shown to give rise to PDACs, the phenotype of the tumour differs depending on the cell of origin (Ferreira et al., 2017). Ductal cells give rise to rapid and invasive PDAC when KRAS mutation is combined with p53, Fbw7 or Pten deletion (Ferreira et al., 2017; Kopp et al., 2018; Lee et al., 2019). However, acinar-derived PDACs have slower progression (Lee et al., 2019). In addition, acinar-derived PDACs often generate PanINs, and express low AGR2 levels. Conversely, given the same oncogenic background, ductal-derived PDAC development is faster and is associated with lower survival and IPMN lesions (Ferreira et al., 2017; Lee et al., 2019; Riva et al., 2018; Yamaguchi et al., 2018). Taken together, this work supports the idea that different progenitors or cells of origin could be linked to PDACs with different prognosis (Fig. 3).

Though less frequent and less aggressive, tumours in the endocrine pancreas have been widely studied. These tumours, known as pancreatic neuroendocrine tumours (PanNETs or PNETs), are initiated in specific hormone-secreting cells of the islets of Langerhans (Batukbhai and De Jesus-Acosta, 2019). They are classified based on the hormone they oversecrete: insulinoma (insulin), glucagonoma (glucagon), somatostatinoma (somatostatin), VIPoma (VIP), gastrinoma (gastrin), PPoma (PP) or non-functioning (no over-secreted hormone) (Fig. 3) (Cives and Strosberg, 2018). These tumours are usually divided into well differentiated NETs, with a low grade of proliferation, and poorly differentiated neuroendocrine carcinomas (NECs), with a high level of proliferation (Choe et al., 2019). NECs and NETs are not only different in prognosis but also show distinct molecular features, suggesting that the different pathologies might come from distinct cells of origin (Scarpa, 2019). Well-differentiated NETs are associated with dysregulation in the PDX1 or NGN3 transcription factors related to endocrine pancreas development (Hermann et al., 2011). Interestingly, it has been shown that NETs may exhibit alternative non-islet cell origins. NETs harbouring mutations in the tumour suppressor gene MEN1 (multiple endocrine neoplasia) have been suggested to be derived from ductal or acinar cells, and possibly from progenitor cells of these compartments after accumulating mutations in MEN1 (Vortmeyer et al., 2004). Although the cell of origin for poorly differentiated NECs is still unknown, the completely different mutations causing NECs indicate that they could be originated from a different cell type (Kloppel, 2017).

As depicted in this section, a complete understanding of the cells originating endocrine tumours and what role they play in normal pancreas function, as well as whether in PDAC any acinar cell can go through ADM or whether it is a pool of progenitor cells that do so, still remains elusive.

## 5 Studying pancreatic progenitors: ES, iPS and organoids models

Because of the inherent challenges that come with studying human development, the molecular regulation of pancreas progenitors was initially studied using murine models. Development of endoderm using mouse embryonic stem cells (mESCs) has served as the base for studying human pluripotent stem cells and their ability to differentiate to endocrine cells (Kubo et al., 2004; Segev et al., 2004; Soria et al., 2000). However, ESC and iPSC technology developed over the last 20 years has greatly contributed to our understanding of the signalling pathways regulating human pancreas progenitors and, in particular, in the generation of β-cells.

By chemically mimicking the different steps which occur during embryonic development of the pancreas, ESCs and iPSCs have been successfully differentiated into pancreas progenitors and β-like cells (D'Amour et al., 2006; Kroon et al., 2008; Pagliuca et al., 2014; Rezania et al., 2014; Rezania et al., 2012; Russ et al., 2015) (Fig. 2B). Initial human ESCs differentiation protocols directing cells towards pancreas progenitors demonstrated the co-existence of two transitory progenitor types: epithelial progenitors and endocrine precursors (D'Amour et al., 2006). The endocrine precursors expressed the embryonic pancreas progenitor markers PDX1, NKX6.1 and NGN3 (Fig. 2B). However, the resulting differentiated cells were comparable to human foetal endodermal/epithelial cells within the pancreas, and showed limited functionality (D'Amour et al., 2006).

Subsequent studies developed better protocols to improve the efficiency of generation and the functionality of β-like cells (Kroon et al., 2008; Pagliuca et al., 2014; Rezania et al., 2012, 2014; Russ et al., 2015). For example, in suspension cultures, more functional pancreatic endocrine cells (NKX2.2+) and pancreatic endodermal cells (NKX6.1+ PDX1+) were generated. In this context, the existence of bi-hormonal cells not expressing PDX1, NKX6.1 and MAFA and positive for ARX, MAFB, NKX2.2 resembled cell fates observed during human foetal development. Interestingly, adding suspension culture as a short last step to culture conditions was enough to yield a higher functionality of β-cells, as the PDX1+ progenitor cells obtained from human ESCs could efficiently rescue diabetes both in mice and rats upon *in vivo* transplantation (Rezania et al., 2012).

Due to their pivotal role in endocrine differentiation, highly efficient differentiation protocols have focused on the development of PDX1+NKX6.1+ progenitor cells, from both human ESCs and iPSCs, using 3D culturing methods such as spinner flasks and orbital shakers (Fig. 2B) (Pagliuca et al., 2014; Russ et al., 2015). Multipotent progenitors obtained from human iPSC lines can be expanded as aggregates and then differentiated over the long term towards glucagon, insulin and somatostatin-expressing cells, similarly to the progenitors without expansion (Konagaya and Iwata, 2019). In addition, several strategies to increase the functionality of the β-cells generated have been described. In one such strategy, enhancing the expression of MAFA in β-like cells that resembled native human islets correlated with a more mature phenotype (Rezania et al., 2014). Furthermore, enrichment for C-peptide+ NKX6.1+ cells in clusters has proven to ultimately increase the yield of functional β-cells (Pagliuca et al., 2014). Interestingly, this protocol was also successfully used to differentiate iPSCs from a type 1 diabetes patient, indicating its potential use in disease modelling and clinical approaches (Millman et al., 2016). While these new strategies have promising potential, further optimization is required to improve functionality of the obtained insulin-producing cells.

The different stages of differentiation from ESCs/iPSCs cells to β-cells have been thoroughly characterised by scRNA-seq. These studies indicated the presence of endocrine progenitors (PDX1+ NKX6.1+ NGN3+), endocrine cells (β-like cells, α-like cells, enterochromaffin-like cells) and one type of SOX9+ non-endocrine cell that develops into acinar, mesenchymal, and ductal cells (Veres et al., 2019). Poly-hormonal cells co-expressing insulin and glucagon were identified as being transitory towards mono-hormonal α-like cells, recapitulating normal human foetal pancreatic development. NGN3+ progenitors were found to give rise to both β-like and enterochromaffin-like cells (Veres et al., 2019). Interestingly, re-aggregation of sorted insulin-expressing clusters resulted in increased functionality of β-cells, suggesting that cell-cell contact could activate key signalling pathways required for β-cell maturation (Veres et al., 2019).

The role these pathways play in cell fate determination has been widely explored using iPSCs/ESCs-to-β-cell differentiation to recapitulate pancreatic development. In addition to the well-known role of the Nodal (Brown et al., 2011; Zorn and Wells, 2009), Sonic Hedgehog (Hebrok et al., 1998), FGF (Wells and Melton, 2000), Notch (Shih et al., 2012), Wnt, and Hippo (Cebola et al., 2015; Rosado-Olivieri et al., 2019; Sharon et al., 2019b) pathways during embryonic development, targeting some of these pathways may increase the efficiency of β-cell generation from iPS or ES cells. In particular, activation of Wnt and Hippo pathways effector YAP have been identified in iPSCs/ESCs-derived multipotent pancreas progenitors that have a high proliferation capacity. Those pathways are suppressed in the differentiating endocrine progenitors *in vitro*, similarly to events occurring during mouse development (Rosado-Olivieri et al., 2019; Sharon et al., 2019b). Proteomic analysis of human pluripotent stem cell-derived pancreatic progenitors identified the Hippo pathway as a key player in cell fate decisions (Loo et al., 2020). Furthermore, chemical inhibition of Wnt and YAP has been shown to increase the yield of endocrine progenitors and β-like cells (Rosado-Olivieri et al., 2019; Sharon et al., 2019b). A more in-depth study of the signalling cues that regulate human multipotent pancreatic progenitors used both human embryos and their equivalent iPSC/ESC-derived cells to study the enhancers that are occupied by the key developmental pancreatic transcription factors HNF1β, ONECUT1, PDX1, GATA6 and FOXA2 (Cebola et al., 2015). The study identified TEAD1 as a new Hippo signalling component critical in pancreas development. TEAD1 and its coactivator YAP were found to be restricted to multipotent progenitor enhancers functioning to increase proliferation during early pancreatic development. This study unravelled a new role of the Hippo pathway in multipotent progenitors both *in vivo* and *in vitro* and suggests that ESCs/iPSCs-derived progenitors are a suitable model to study mechanisms and gene regulation during human pancreas development.

Besides ESCs and iPSCs, organoid technology has further facilitated the study of cell plasticity from an adult progenitor perspective. The progenitor properties can be demonstrated by culturing adult tissue-derived cells as three-dimensional cell clusters (organoids), that recapitulate features of the originating organ (Sato et al., 2011). Organoid culture systems were first established to study intestinal stem cells (Barker et al., 2007; Sato et al., 2011) and then extended to other endodermal tissues (Fatehullah et al., 2016).

Although not highly active *in vivo*, specific populations of pancreatic cells can show expansion and multipotency *in vitro*. While classic pancreatic organoids do not harbour all pancreatic cell types in basic culture conditions, the potential of pancreatic organoids as a platform has been already established for developmental, disease modelling and cancer related studies (Azzarelli et al., 2017; Boj et al., 2015; Chio et al., 2016; Dossena et al., 2020; Huang et al., 2015; Ohlund et al., 2017), as well as host-pathogen interaction in the context of infection (Bartfeld et al., 2015). Additionally, different *in vitro* conditions have been proven to induce insulin expression in some of the cells (Loomans et al., 2018).

Organoids derived from pancreatic tissue express typical ductal markers (SOX9, KRT19, MUC1), indicating that progenitor potential is associated with a ductal phenotype (Fig. 3) (Boj et al., 2015; Huch et al., 2013; Lee et al., 2013; Yatoh et al., 2007). When non-endocrine EpCAM+ pancreatic cells were sorted and cultured in 3D organoid conditions, the ones capable of long-term expansion were SOX9+ (Huch et al., 2013), further indicating that the ductal compartment could be the one with progenitor features *ex vivo*. Taking advantage of the similarities with the neural system, other groups have used the surface marker CD133 to isolate and expand pancreatic organoids (Immervoll et al., 2008; Uchida et al., 2000). While this population cannot be differentiated *in vitro* unless the β-cell specific factors MAFA, PDX1 and NGN3 are overexpressed via adenoviral infection, the combination of CD133+ and CD71-low expression resulted in cells successfully able to yield different lineages *in vivo* (Jin et al., 2013, 2014; Lee et al., 2013; Sugiyama et al., 2007). More recently, high ALDH1B1-expressing ductal (centroacinar) cells efficiently formed organoids that could be maintained for long periods *in vitro* (Loomans et al., 2018; Rovira et al., 2010), suggesting not only that ALDH1B1+ might represent a selection marker for progenitor cells able to expand and differentiate into insulin producing cells, but also that this gene might be required for this outcome (Mameishvili et al., 2019).

Ductal-derived organoids generated from adult pancreas are often described as spheres, with no further organization such as tip-trunk compartmentalization or spontaneous differentiation to endocrine/acinar cells (Georgakopoulos et al., 2020; Lee et al., 2013). However, since the establishment of the pancreatic ductal organoid model, several protocols have been developed in order to maintain the potency/plasticity of the spheres as well as attempting to differentiate them to endocrine cells (Fig. 3).

In addition to ductal organoids, 3D cultures have also been achieved using other pancreatic cell types such as acinar and islet cells. Multicolour lineage-tracing and organoid-formation assays of acinar cells led to the identification of a population with progenitor-like features, able to transdifferentiate into a ductal cell type (Wollny et al., 2016). This metaplastic transformation resulted in the loss of amylase expression and KRT19 activation, confirming that a subset of acinar cells might be “primed” for ADM and long-term expansion *ex vivo* (Houbracken et al., 2011). However, these acinar progenitors might be involved in ADM induced tumour formation (see section 4). A recent study has demonstrated that Procr+ islet cells, adult endocrine progenitors derived from Ngn3+ embryonic progenitors, can generate islet-like organoids *ex vivo*, which have the ability to reverse hyperglycemia when reintroduced into a diabetic host (Wang et al., 2020). The ability to propagate *in vitro* was enhanced upon co-culture with endothelial cells, forming organoids that could be expanded through passaging (Fig. 3). Interestingly, organoids left to grow without passaging spontaneously generated islet-sized structures that retained a remarkable functionality to control glucose homeostasis when transplanted into diabetic mice (Wang et al., 2020a). While the composition of these organoids has not been fully elucidated yet, newly developed technologies will allow a comprehensive characterization of cell types in the next few years.

## 6 Identifying stem/progenitor cells: scRNA-seq technology

One of such newly developed technologies is the increasingly utilised scRNA-seq. Although, when complemented with experimental *in vitro* and *in vivo* validation, this technique is of great value, when considered in isolation it presents limitations that could compromise the biological relevance of the findings. These include the inaccurate cell identity assignment by comparing cells with pre-existing transcriptional profiles, the use of inadequate sequencing depth which could result in inefficient detection of low expressed transcripts, or technical limitations when processing the tissue that could result in under representation of cells in the datasets. However, though not devoid of challenges (Chen et al., 2019), RNA-seq technology has enabled identification of novel cell types, refined the classification of cell-specific markers, and provided insights into cellular heterogeneity in the pancreas (Baran-Gale et al., 2017).

**Fig. 4:**
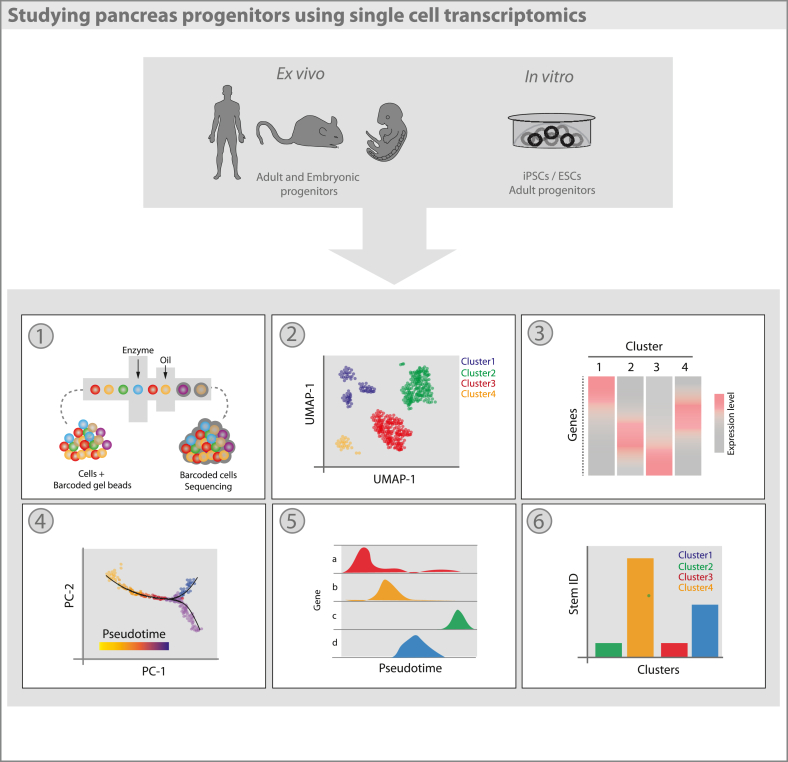
Transcriptomic profiling of human and embryonic tissues, experimental animal models and patient-derived cell lines via scRNA-seq enables the study of pancreas progenitors. (1) Single-cell suspensions enter high throughput microfluidic systems (e.g. 10X Chromium, Drop-Seq) allowing thousands of cells to be processed and sequenced. (2) Cells undergo dimensionality reduction and are clustered based upon expression profiles – represented via UMAP or t-SNE - enabling the identification of novel cell types and investigation of cellular heterogeneity. (3) Following clustering, differential expression analysis reveals changes in gene expression across cell types. (4) Prediction of cell trajectories can be inferred based upon changes in gene expression over a ‘pseudo’ time-course. Cells are ordered in a 2D space based upon the closeness of their expression pro_les. Overlay of a minimal spanning tree (MST) identifies the longest continual path linking these cells – uncovering cell lineages. (5) Individual trajectories can be dissected and changes in specific gene expression changes plotted in both a supervised and unsupervised manner (6) The development of algorithms (e.g. StemID, SCENT) has enabled the prediction of cell clusters with high potency, stem-like features. Used in conjunction with pseudotime analysis, these algorithms can infer a starting point of differentiation trajectories, as well as identifying novel stem cells in adult tissues.

In recent years, numerous scRNA-seq studies of both human and mouse pancreatic islets have been published (Baron et al., 2016; Enge et al., 2017; Muraro et al., 2016; Regev et al., 2017; Tabula Muris et al., 2018). Studies involving the embryonic pancreas as well as iPSC and ESC-derived endocrine cells have provided clues to the transcriptomic processes underpinning pancreatic development (Krentz et al., 2018; Sharon et al., 2019a; Veres et al., 2019). Furthermore, studies using patient derived samples have enabled analyses of transcriptomic changes in the context of metabolic disease and cancer; providing potential therapeutic targets for the respective diseases (Aier et al., 2019; Peng et al., 2019) (Fig. 4).

Much of our understanding of pancreatic development and homeostasis comes from embryonic and mouse lineage tracing studies. These techniques have now been used in conjunction with powerful single cell transcriptomics to provide a clearer picture of the processes governing organogenesis and self-renewal (Byrnes et al., 2018). scRNA-seq analyses at different timepoints in the iPSC/ESC to β-cell differentiation process have been used to assess changes in the expression profiles during the differentiation steps (Krentz et al., 2018; Veres et al., 2019). While these data suggest that *in vitro* differentiation protocols closely recapitulate the stages of pancreatic embryonic development, there is still much to clarify as different studies have shown different resolution and identified different populations (Mawla and Huising, 2019). Further insights into heterogeneity of the cell types generated, along with the molecular and temporal control of cell fate decisions is needed to further our knowledge of pancreatic cell biology.

One longstanding question in the field of diabetes and pancreatic cell biology is whether pancreas-resident progenitors can give rise to insulin-producing cells. There have been many postulated sources of progenitors in the adult pancreas (Bonner-Weir et al., 2008; Grun et al., 2016; Qadir et al., 2020). By applying scRNA-seq it is possible to observe immature, double hormone-positive endocrine cells within the adult islet and gain information into their relative similarity with extra-islet cells (Teo et al., 2018; Wang et al., 2020). This population was however barely observed before (Herrera, 2000), opening the question as to whether it was a matter of technology resolution as opposed to accuracy (Katsuta et al., 2010). Furthermore, to date the majority of pancreatic single cell data is restricted to islets, due to difficulties in maintaining the integrity of ducts and acinar cells when dissociating the pancreas. As a consequence, further technological advancements (e.g., single-nucleus RNA-seq) are under development to allow a full and more precise picture of the single cell transcriptome of the pancreas (Ding et al., 2020). These developments could shed further light on whether progenitors in the different pancreatic compartments exist and what their function is during homeostasis and disease.

Given the rising interest in organogenesis and disease modelling through the use of organoids, identification of a stem cell population would yield organoids derived from a less heterogeneous pool of cells, with greater organoid forming capacity and greater propensity for endocrine differentiation (Fig. 4). An example is the work by Stanescu and collaborators, which identified SLC38A5 as potential marker for alpha lineage specification (Stanescu et al., 2017). With the increasing resolution and high throughput of scRNA-sequencing platforms (e.g.,10X Chromium, inDrop, Fluidigm C1) it is now possible to capture a sufficient cross-section of cells at varying differentiation propensities to enable comparison of individual transcriptomes and identify cell states (Baran-Gale et al., 2017). However, consistent detection of low abundance transcripts resulting in count drop-outs is still a challenge, rendering this an imperfect tool for the time being. Trajectory analysis can place and order cell profiles upon a trajectory, enabling changes in gene expression over a ‘pseudotime’ to be assessed. Tools to evaluate these pseudo-temporal changes have been developed, enabling investigation of differentiation trajectories (Monocle, SLICER, TSCAN, Slingshot etc.) (Ji and Ji, 2016; Street et al., 2018; Trapnell et al., 2014; Welch et al., 2016). In order to identify stem cells within a given dataset, cell profiles can be used to reconstitute a lineage tree showing the topology of differentiation trajectories in a pseudotemporal model (Trapnell et al., 2014). This description of the dynamic state of cells can be used to predict putative stem cells from the population as a whole (Teschendorff and Enver, 2017). Cells can further be assessed for transcriptome uniformity gradients and subsequent branch number to provide a pluripotency score. These scores can be used to infer the ordering of cells on a lineage tree and give confidence to subsequent pseudo-trajectory analyses (Teschendorff and Enver, 2017). Work by Alexander von Oudenaardenn's group has yielded algorithms to identify rare cell (RaceID) and stem cell (StemID) populations. This later method has been shown to predict multipotent cell populations within the adult human pancreas which require further investigation (Grun et al., 2016; Muraro et al., 2016) (Fig. 4). Similar work by the Trapnell Lab, has developed single cell entropy scoring algorithm (SCENT) to predict potential progenitor cells (Teschendorff and Enver, 2017).

A key goal in cell biology is to delineate the mechanisms by which cells form tissues and organs, and how these processes are modulated both in healthy and disease settings. Understanding transcriptomic changes occurring between these dynamic cell states could allow researchers to gain better insight on the progenitor cells within the pancreas, as well as achieve manipulation of the molecular mechanisms governing pancreatic cell fates in order to improve therapeutic strategies.

## 7 Concluding remarks

Pancreas progenitor research has been classically focused on the embryonic stage both in human and in mouse. Embryonic progenitors have been fully characterised and their potential and signalling pathways regulating their fate decisions are well known. A complete hierarchy of cell lineages has been elucidated and has served as a guide to differentiate pluripotent stem cells to β-cells. However, the existence of progenitors beyond the embryonic stage in the pancreas has been questioned over the years. While it is clear that the adult differentiated cells retain some proliferative capacity, the existence of stem/progenitor cells in the pancreas is still under debate.

The latest work using murine models suggests that the pancreas could contain some progenitor-like cells that sustain basal homeostasis (Dominguez-Bendala et al., 2019; Mameishvili et al., 2019; Wang et al., 2020). Furthermore, regeneration has been suggested in the rodent pancreas after challenges like inflammatory cytokines, tissue damage (pancreatic duct ligation), loss of β-cells, oncogenic stress and forced expression of proteins involved in embryonic development of the pancreas. However, whether these properties are relevant in the human pancreas is still not known. Much work is needed to address to what extent the regenerative potential of the human pancreas shares similarities with that of the murine pancreas.

Culturing adult and ESCs/iPSCs-derived progenitors using organoid technology has allowed the molecular characterization of pancreas progenitors *ex vivo* while providing a platform to identify new ways of inducing endocrine differentiation. Tumour organoids have also been derived to study cancer cells in the pancreas and for personalised drug screenings. Although the full composition of organoids has not been fully elucidated yet, new technologies (single cell transcriptomics/proteomics) will allow a comprehensive characterization in the near future. The rise of inference techniques based on scRNA-seq has been pivotal to delineating novel lineage trajectories and identifying putative *in vivo* progenitors, despite their limitations. Transcriptomics techniques will greatly contribute to future studies aimed at obtaining a full picture of organoid-forming cells and interrogating the function of those cells in normal pancreatic physiology and/or regeneration both in mouse and humans.

While in the last few years the previously unknown regenerative function of the pancreas has started to be uncovered in rodents and humans, advances in technologies will allow further insight into putative progenitors and signaling pathways in the human pancreas, which could have high therapeutic potential.

## Authors contributions

M.A.F – conceptualization, coordination revisions, writing review, editing; S.P.A - conceptualization, coordination revisions, writing review, editing; A.M.C – writing review, editing; T.M – writing review, editing; C.L – writing review, editing; R.M – writing review; R.S – supervision, conceptualization, writing review.
